# Non-Invasive Measurement of Hemoglobin: Assessment of Two Different Point-of-Care Technologies

**DOI:** 10.1371/journal.pone.0030065

**Published:** 2012-01-06

**Authors:** Etienne Gayat, Jérôme Aulagnier, Emmanuel Matthieu, Mireille Boisson, Marc Fischler

**Affiliations:** 1 Department of Anesthesia, Foch Hospital, Suresnes, France; 2 Clinical Epidemiology and Biostatistics, INSERM U717, Saint-Louis Hospital, Paris, France; 3 Emergency room, Foch Hospital, Suresnes, France; 4 Department of Biology, Foch Hospital, Suresnes, France; University of Colorado Denver, United States of America

## Abstract

**Background:**

Measurement of blood hemoglobin (Hb) concentration is a routine procedure. Using a non-invasive point-of-care device reduces pain and discomfort for the patient and allows time saving in patient care. The aims of the present study were to assess the concordance of Hb levels obtained non-invasively with the Pronto-7 monitor (version 2.1.9, Masimo Corporation, Irvine, USA) or with the NBM-200MP monitor (Orsense, Nes Ziona, Israel) and the values obtained from the usual colorimetric method using blood samples and to determine the source of discordance.

**Methods and Findings:**

We conducted two consecutive prospective open trials enrolling patients presenting in the emergency department of a university hospital. The first was designed to assess Pronto-7™ and the second NBM-200MP™. In each study, the main outcome measure was the agreement between both methods. Independent factors associated with the bias were determined using multiple linear regression. Three hundred patients were prospectively enrolled in each study. For Pronto-7™, the absolute mean difference was 0.56 g.L^−1^ (95% confidence interval [CI] 0.41 to 0.69) with an upper agreement limit at 2.94 g.L^−1^ (95% CI [2.70;3.19]), a lower agreement limit at -1.84 g.L^−1^ (95% CI [-2.08;-1.58]) and an intra-class correlation coefficient at 0.80 (95% CI [0.74;0.84]). The corresponding values for the NBM-200MP™ were 0.21 [0.02;0.39], 3.42 [3.10;3.74], -3.01 [-3.32;-2.69] and 0.69 [0.62;0.75]. Multivariate analysis showed that age and laboratory values of hemoglobin were independently associated with the bias when using Pronto-7™, while perfusion index and laboratory value of hemoglobin were independently associated with the bias when using NBM-200MP™.

**Conclusion:**

Despite a relatively limited bias in both cases, the large limits of agreement found in both cases render the clinical usefulness of such devices debatable. For both devices, the bias is independently and inversely associated with the true value of hemoglobin.

**Trial Registration:**

ClinicalTrials.gov NCT01321580 NCT01321593

## Introduction

After the introduction of pulse oximetry [1] which dramatically improved patient care, particularly in acute conditions in the emergency room (ER), the recent development of devices allowing non-invasive and almost immediate measurement of hemoglobin (SpHb) is promising.

Indeed, blood hemoglobin is routinely assessed mainly for two purposes, to diagnose anemia, and then to pursue more invasive testing, and to assess the need for blood transfusion. The cut-off values leading to the diagnosis of anemia are widely accepted [2], [3] while the need for transfusion is decided after putting the hemoglobin value in the perspective of the clinical context of the patient. In the emergency department, as in other settings, laboratory measurement of hemoglobin requires transport of samples thus delaying the process. The potential improvement in patient care with a non-invasive solution for measuring hemoglobin could be important as it gives the result more rapidly, decreases exposure to potential biohazards, and finally reduces pain and discomfort to the patient.

To date, four peer-reviewed publications [4], [5], [6], [7] have assessed non-invasive solutions for hemoglobin measurement. All of them assessed the same technology using multi-wavelength pulse CO-oximeters, namely the monitor Radical-7™ (Masimo Corporation, Irvine, USA). The results of the four studies were discordant and in three cases [4], [5] a relatively large discrepancy was reported between non-invasive measurements (SpHb) and classic measurement (Hb-Lab). Since these reports, in order to improve the accuracy of the non-invasive measurements, new monitors and probes have been developed. Moreover, another monitor has recently been released. This new device, namely the NBM-200MP™ (Orsense, Nes Ziona, Israel), uses differential light absorption before and after blood flow obstruction in a finger to determine hemoglobin level non-invasively. To date, no study assessing this device has been published.

Accordingly, we report the results of two subsequent studies using the same design, assessing two devices, the monitor Pronto-7™ (version 2.1.9, Masimo Corporation, Irvine, USA) and the monitor NBM-200MP™. The two studies are hereafter referred to as the “Masimo-Study” and the “Orsense-Study”. The aims of the studies were i) to assess the concordance of hemoglobin levels obtained non-invasively with the values obtained from the usual colorimetric method in the hospital laboratory and using blood samples and ii) to determine the source of errors in the measurements.

## Materials and Methods

Design and data analysis were strictly similar for the “Pronto-Study” (ClinicalTrials.gov Identifier: NCT01321580) and the “Orsense-Study” (ClinicalTrials.gov Identifier: NCT01321593) which were conducted consecutively (from December 15^th^ 2010 to February 15^th^ 2011 for the first and from February 15^th^ 2011 to March 31^st^ for the second).

These studies were conducted in accordance with the STARD guidelines (STAndards for the Reporting of Diagnostic accuracy studies) [8].

### Participants

These prospective open studies, performed in the emergency department of a university hospital, were approved by the Ethics Committee (CPP Ile-de-France VIII). Consecutive patients examined by the same senior emergency nurse and requiring a hemoglobin measurement were enrolled after they gave their informed written consent. Only the laboratory results were used for subsequent patient care.

### Study protocol and measurements

Investigators recorded the hemoglobin level determined non-invasively by the Monitor Pronto-7™ (version 2.1.9) with the Rainbow^®^ 4D DC sensor (revision B) (Masimo Corporation, Irvine, USA) or by the monitor NBM-200MP™ (Orsense, Nes Ziona, Israel), with the probe placed on the patient's finger, while a nurse collected a venous blood sample in a EDTA (EthyleneDiamineTetraacetic Acid) tube, which was sent immediately to the hematology laboratory for hemoglobin measurement by the ADVIA^®^ 2120 (Siemens Medical Solutions Diagnostics, Zurich, Switzerland). Three consecutive values of SpHb were collected; the first two minutes after the probe had been placed on the patient's finger and two others after two minute intervals, all the measurements were obtained over a 6 minute period. Only one simultaneous blood sampling was performed for each patient. The measure of hemoglobin level by the ADVIA^®^ 2120 method was considered to be the gold-standard measure.

At the same time, body temperature, systolic and diastolic blood pressure, peripheral oxygen saturation, perfusion index (displayed on the Pronto-7™ monitor or on the NBM-200MP™ monitor) and heart rate were collected. Of note, the Perfusion Index (PI) of the Pronto-7 monitor provides a numeric indication of the pulse strength at the measurement site. It is a calculated percentage between the pulsatile signal and non-pulsatile signal of arterial blood moving through the site. A similar index exists for the NBM-200MP. However, concerning the Orsense device, unlike standard sensors, the NBM sensor is located on the root of the finger, where perfusion is better than in the fingertip and as the measurement is based on occlusion spectroscopy technology it is thought to rely less on the pulse signal.

During the procedure, the anxiety and the pain of the patients were assessed using in both cases a visual analog scale (VAS). The VAS was graphically represented as a horizontal line, 100 millimeter (mm) in length, with word descriptors at each end (“No pain” and “Very severe pain” for pain assessment and “I feel totally relaxed” and “I feel highly anxious” for the anxiety assessment”). The patient marked on the line the point that they felt represented their perception of their current state. The VAS score was determined by measuring in mm from the left hand end of the line to the point that the patient marked. The evaluation was performed 2 min after the placement of the probe on the patient's finger and 2 min after the blood sampling.

### Statistical analysis

Results are expressed as median and first and third quartiles [Q1 to Q3] or counts and percentages. Comparison of values obtained by the two methods was performed with a paired Student t test. The individual values of SpHb and perfusion index (PI) were defined as the mean of the three measurements. For the concordance analysis, in both studies, we used the mean of the three consecutive non-invasive measurements to define the value of SpHb.

For agreement between the two hemoglobin determination methods (invasive and non-invasive), a Bland–Altman analysis was applied calculating bias as the mean difference between both methods and limits of agreement as the range in which 95% of the differences between the two methods are expected to lie [9]. The intra-class correlation coefficient (ICC) between the two methods was also estimated.

Multivariate linear regression was used to determine a set of variables independently associated with bias. Bias was defined as the absolute difference between the two measurements and was expressed in g/dL. For continuous covariates, the linearity assumption was checked using additive regression models with splines. Model goodness-of-fit was assessed by examination of residuals (quantile to quantile plots and residuals versus fitted plots).

The percentage of variation over the three non-invasive measurements was determined. First, the variation was calculated for each of the three measurements, then the highest value was used to define the individual coefficient of variation of the non-invasive measurement.

The within-subject coefficient of variation of each noninvasive and invasive technique was calculated by analyzing 15 different samples in duplicate. The coefficient of variation is defined as the ratio of the standard deviation of the absolute difference between the duplicates divided by the mean of the average value of the duplicate [10].

According to the methods developed for reliability study [11], the calculated sample size was 270. Based on previous work on hemoglobin assessment [12], the hypothesis to obtain this sample size was an expected ICC at 0.88 with a width of the confidence interval at 0.1. Given a rate of inconclusive measurements or lost to follow-up of 10%, the number of patients to include was 300 in each study.

Comparisons of pain and anxiety VAS scores were also performed using non-linear mixed effect models, in order to take into account the intra-subject correlation.

A two-sided p-value of 0.05 was considered significant. All analyses were performed using R 2.10.1 statistical software (The R Foundation for Statistical Computing, Vienna, Austria).

## Results

### Patients' characteristics

**Figure 1:**
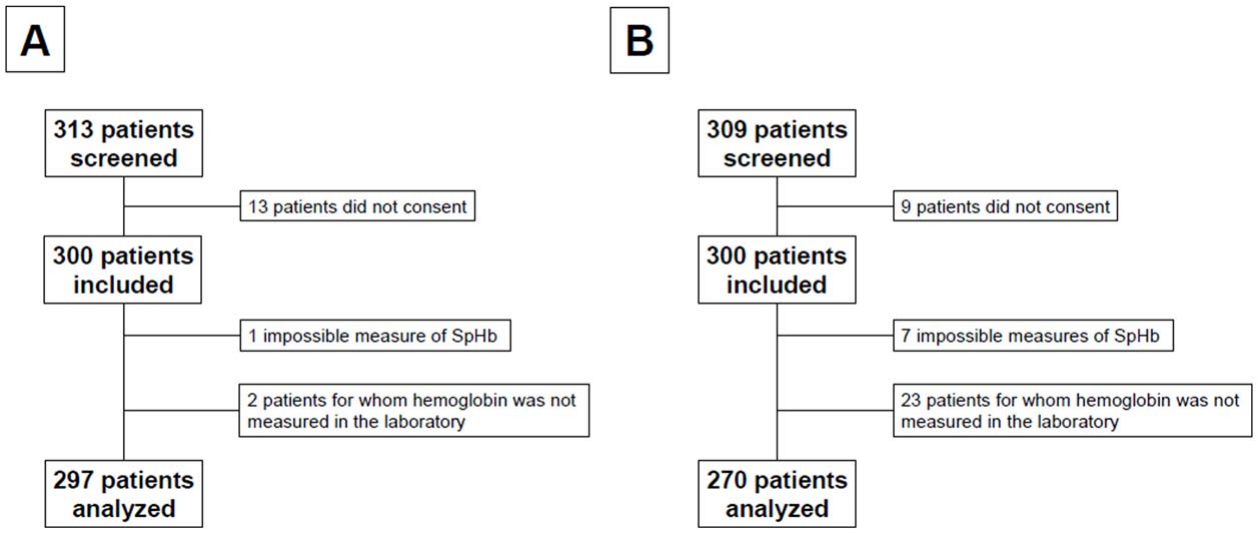
Flow chart of the studies (Pronto-7™ on panel A and Orsense on panel B).

**Figure 2:**
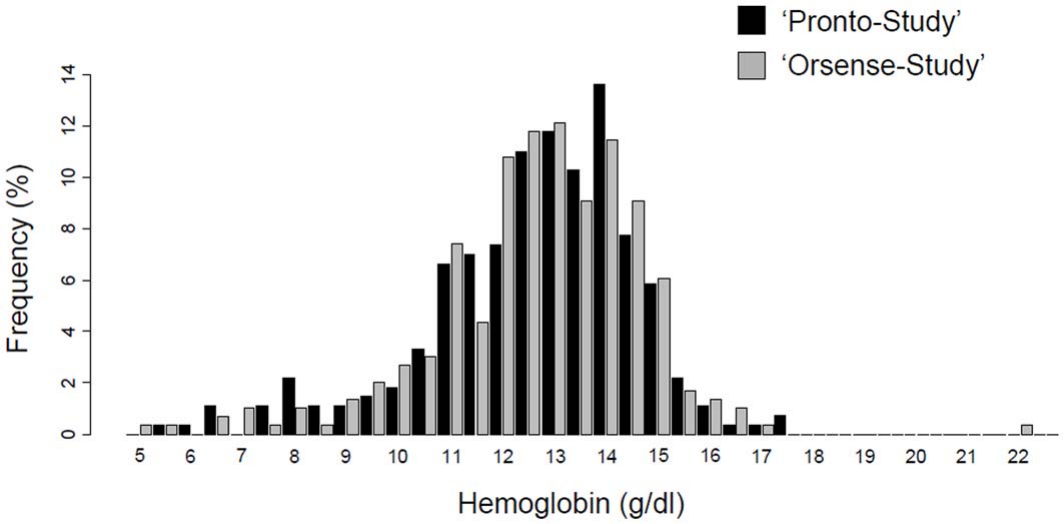
Distribution of hemoglobin value in the two populations studied. As depicted in the figure, the overlap of hemoglobin values between the two populations studied is almost complete all along the range of hemoglobin values.

Three hundred patients were prospectively included in each study. The flow chart of the studies is shown in **Figure 1**. Patient characteristics are described in **Table 1**. Principal causes for admission to the emergency ward were abdominal pain (21%), thoracic pain (13%), dyspnea (12%) and sepsis (8%). Seven per cent of the patients were admitted because of active, or recent history of, bleeding. None of the patients' characteristics were different between the two studies. Non-invasive hemoglobin values could not be obtained in 5 patients when using the Pronto-7 hemoglobin-meter and in 2 patients when using the NBM-200MP™. Laboratory measure was not obtained in 23 patients in the Pronto-Study and in 1 patient in the Orsense-Study, because of discharge from the ER prior to the blood sample. As depicted in **figure 2**, the distribution of hemoglobin levels in the two studied populations was similar even for the extreme values.

**Table 1:** Patient characteristics (continuous data are expressed as mean ± SD or median [interquartile range]).

	All patients(n = 569)	Pronto-7™(n = 272)	Orsense™(n = 297)	p value
**Age (years)**	57 (42 to 74)	57 (43 to 75)	58 (41 to 74)	0.68
**Male gender**	297 (52.2)	140 (51.5)	157 (52.9)	0.74
**Height (cm)**	170 (162 to 175)	170 (162 to 175)	170 (162 to 175)	0.8
**Weight (kg)**	70 (60 to 80)	70 (59 to 81.8)	68.5 (60 to 80)	0.15
**Reasons for ER admission**				0.13
Abdominal pain	122 (21)	48 (18)	74 (25)	
Fatigue	36 (6)	17 (6)	19 (6)	
Bleeding	39 (7)	20 (7)	19 (6)	
Chest pain	74 (13)	36 (13)	38 (13)	
Dyspnea	70 (12)	38 (14)	32 (11)	
Faintness	38 (7)	17 (6)	21 (7)	
Other neurological disorders	55 (10)	21 (8)	34 (11)	
Other	73 (13)	45 (17)	28 (9)	
Infection	40 (7)	22 (8)	18 (6)	
Trauma	20 (4)	8 (3)	12 (4)	
Anemia	2 (0)	0 (0)	2 (1)	
**Hemodynamics at admission**				
SBP (mmHg)	133 (114 to 150)	134 (116 to 151)	132 (113.8 to 148)	0.32
DBP (mmHg)	81 (72 to 90)	81 (72 to 90)	81 (72 to 90)	0.51
HR (bpm)	86 (75 to 99)	87 (76.5 to 101)	85 (73 to 98)	0.089
SpO2 (%)	98 (96 to 99)	98 (96 to 99)	98 (96 to 99)	0.19
**Hemoglobin measured in the laloratory (g/dL)**	13.2 (11.9 to 14.3)	13.2 (11.9 to 14.3)	13.2 (12.1 to 14.2)	0.73

(See text for details).

(SBP: systolic blood pressure. DBP: diastolic blood pressure. HR: heart rate. SpO2: pulse-oximeter saturation)

### Concordance between invasive and non-invasive measurements

**Figure 3:**
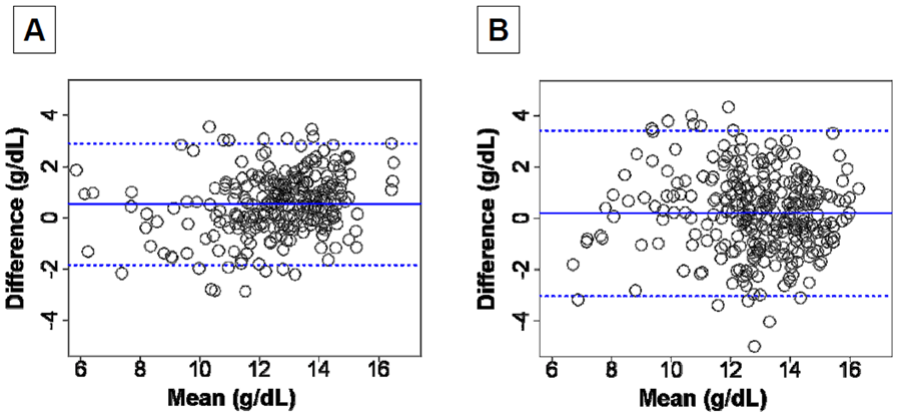
Bland and Altman graphical representation of the concordance between the laboratory value of hemoglobin and SpHb. Results for the Pronto-Study are displayed on the panel A and for the Orsense-Study on the panel B. The plain horizontal blue line represents the mean bias and the dashed horizontal blue lines represent the upper and the lower limits of agreement.

The Bland-Altman graphical representation of the concordance is reported in **Figure 3**. **Table 2** depicts the concordance parameters of the two studies. For Pronto-7™, the absolute mean difference was 0.56 g.L^−1^ (95% confidence interval [CI] 0.41 to 0.69) with an upper agreement limit at 2.94 g.L^−1^ (95% CI 2.70 to 3.19), a lower agreement limit at -1.84 g.L^−1^ (95% CI -2.08 to -1.58) and an intra-class correlation coefficient at 0.80 (95% CI 0.74 to 0.84). The corresponding values for the NBM-200MP™ monitor were 0.21 [0.02; 0.39], 3.42 [3.10; 3.74], -3.01 [-3.32; -2.69] and 0.69 [0.62; 0.75].

**Table 2:** Concordance parameters of the two methods.

	Pronto-7™	Orsense™
**Bias [95% CI]** (g/dL)	0.56[0.41; 0.69]	0.21 [0.02; 0.39]
**Upper limit of agreement** **[95% CI]** (g/dL)	2.94[2.70; 3.19]	3.42 [3.10; 3.74]
**Lower limit of agreement** **[95% CI]** (g/dL)	-1.84[-2.08; -1.58]	-3.01 [-3.32; -2.69]
**ICC coefficient** **[95% CI]** (g/dL)	0.80 [0.74; 0.84]	0.69 [0.62; 0.75]
**Coefficient of variation (CV)**	3.5%	5.9%

(ICC: intra-class correlation. CI: confidence interval).

### Independent predictors of bias

In univariate analysis, age, diastolic blood pressure and the hemoglobin value when assessed in the laboratory were significantly associated with the bias associated with Pronto-7™. Similarly, in univariate analysis, gender, heart rate, body temperature, perfusion index and the hemoglobin value when assessed in the laboratory were significantly associated with the bias associated with Orsense™ (**Table 3**). Multivariate analyses led to identification of two independent factors associated with the bias for Pronto-7™: age and true value of hemoglobin (mean difference [95% CI]: -0.14 [-0.20; -0.08 ] for 10 years and -0.32 [ -0.38; -0.26 ] for 1 g/dL, respectively). Similarly, perfusion index and true value of hemoglobin were both independently associated with the bias of the measurement of Orsense™ (mean difference [95% CI]: 0.339 [0.339-0.340] for 1 unit and -0.998 [-0.997;-1.000] for 1 g/dL, respectively).

**Table 3:** Factors associated with the bias.

	Pronto-7™		Orsense™	
	Effect on bias[95% CI]	p value	Effect on bias[95% CI]	p value
**Age (for 10 years)**	-0.08 [ -0.15 ; -0.0 ]	0.0288	0 [ -0.01 ; 0 ]	0.2827
**Gender**	0 [ -0.29 ; 0.28 ]	0.9808	0.46 [ 0.1 ; 0.83 ]	0.013
**SBP (mmHg)**	0 [ -0.01 ; 0 ]	0.0969	0 [ -0.01 ; 0 ]	0.458
**DBP (mmHg)**	-0.01 [ -0.02 ; 0 ]	0.0031	0 [ -0.01 ; 0.01 ]	0.7281
**HR (bpm)**	0.01 [ 0 ; 0.01 ]	0.071	0.01 [ 0 ; 0.02 ]	0.0359
**SpO2 (%)**	0.03 [ -0.02 ; 0.08 ]	0.3176	0 [ -0.03 ; 0.02 ]	0.7687
**Body temperature (**°**C)**	0.07 [ -0.1 ; 0.25 ]	0.4106	0.3 [ 0.06 ; 0.54 ]	0.0167
**Perfusion index**	-0.01 [ -0.05 ; 0.02 ]	0.3598	0.11 [ 0.08 ; 0.14 ]	< 0.0001
**Hb-Lab**	-0.3 [ -0.36 ; -0.24 ]	< 0.0001	-0.29 [ -0.38 ; -0.21 ]	< 0.0001

(CI: confidence interval. SBP: systolic blood pressure. DBP: diastolic blood pressure. HR: heart rate. SpO2: pulse-oximeter saturation. Hb-Lab: hemoglobin value obtained invasively. PI: perfusion index. VAS: visual analogical scale).

**Figure 4:**
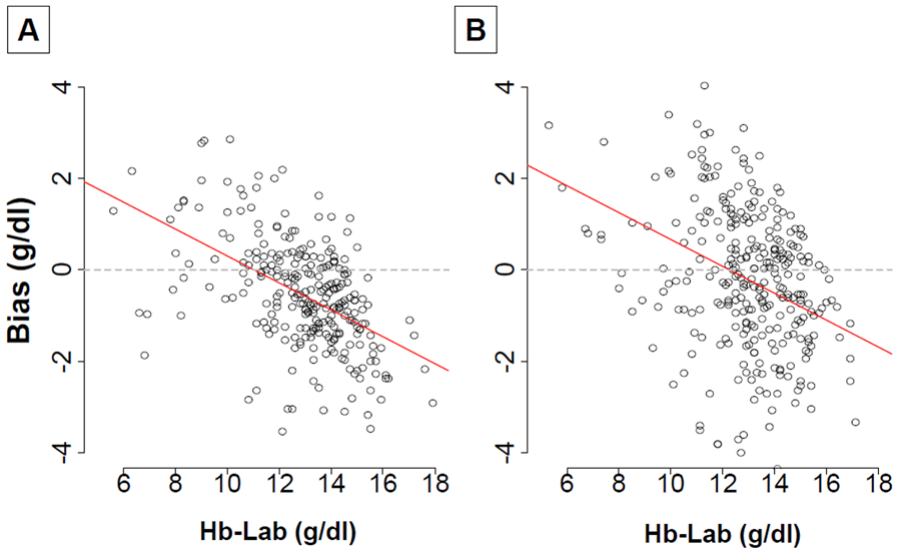
Association between true values of hemoglobin and bias. Results for the Pronto-Study are displayed on the panel A and for the Orsense-Study on the panel B. The red line represents the linear regression relationship between the true value of hemoglobin and the bias. There is an inverse correlation between hemoglobin and bias in both studies (Spearman correlation coefficients at - 0.51, p <0.0001 and -0.37, p<0.0001 for the Pronto-Study and the Orsense-study, respectively).

**Figure 4** depicts the inverse linear relationship between the bias and the true value of hemoglobin found in both studies (Pearson's correlation coefficient at -0.51, 95% CI [-0.59; -0.41], p<0.0001 for the Pronto-Study and at -0.37, 95% CI [-0.46; -0.26], p<0.0001for the Orsense-Study).

**Figure 5:**
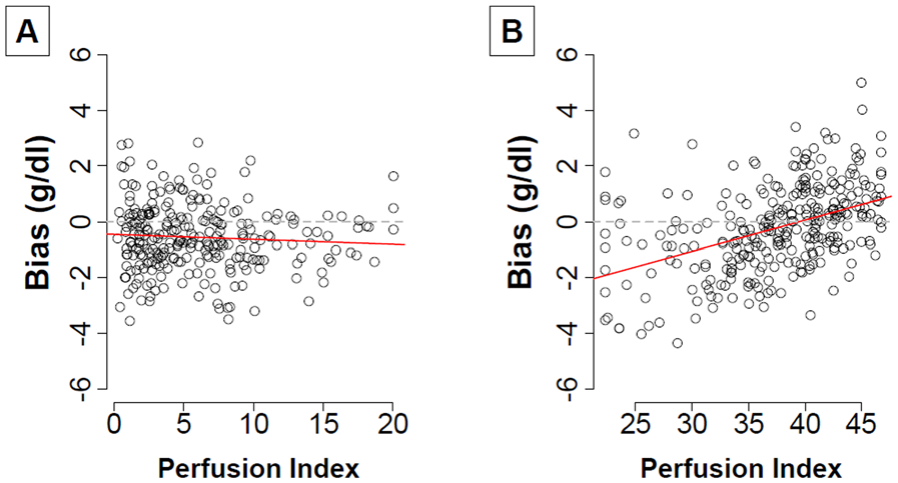
Association between perfusion index and bias. Results for the Pronto-Study are displayed on the panel A and for the Orsense-Study on the panel B. The red line represents the linear regression relationship between the perfusion index and the bias. Perfusion index is linearly correlated with the bias in Orsense-Study but not in the Pronto-Study (Spearman correlation coefficients at - 0.06, p = 0.36 and 0.42, p<0.0001 for the Pronto-Study and Orsense-study, respectively).

Similarly, **Figure 5** illustrates the relationship between bias and perfusion index. Although there is no significant relationship between PI and bias for the Pronto-Study, there is a linear relationship between bias and PI for the Orsense-Study. Concerning the PI, the Pearson's correlation coefficient with the bias were -0.06 [-0.18; 0.05] (p = 0.27) and 0.42 [0.321; 0.51] (p <0.001) for Pronto-7™ and Orsense™ respectively.

### Repeatability of the measurements

**Figure 6:**
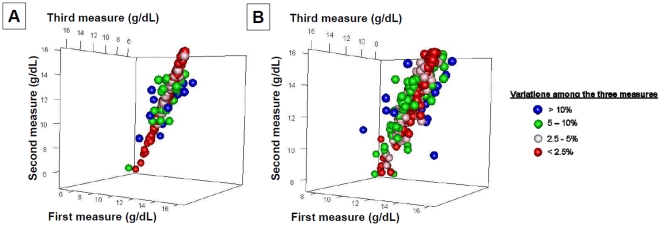
Three-dimensional representation of the variability of the measures obtained with the two monitors. Results for the Pronto-Study are displayed on the panel A and for the Orsense-Study on the panel B. The coordinates of each point are the three consecutive measures. The graphical representation shows that the Orsense™ monitor seems to be associated with a higher variability compared to the Pronto-7™. This is confirmed by the fact that variations among the three consecutive measurements was lower than 2.5% in 71% of the cases when using the Pronto-7™ monitor but only in 47% of the cases when using the Orsense™ monitor

The within-subject coefficients of variation were 1.3% for the ADVIA 2120, 3.5% for the Pronto 7™ and 5.9% for the NBM-200MP™. **Figure 6** depicts in three dimensions the difference between the three non-invasive determinations of the hemoglobin value in each patient for the two devices. For Pronto-7™, the mean percentage of variation was 2.7%±3% and variation was higher than 10% in 11 (4%) patients and lower than 2.5% in 194 (71%) patients. The corresponding values for NBM-200MP™ were 5.1%±4.1% for the mean percentage of variation. Variation was higher than 10% in 18 (6%) patients and lower than 2.5% in 139 (46%) patients.

### Anxiety and pain assessment

The pain and anxiety VAS scores were significantly lower with the non-invasive measurement in both studies. The differences for pain on the VAS scale were 1.62 [1.33; 1.91] (p<0.0001) and 1.62 [1.33; 1.91] (p<0.0001) in the Pronto-Study and the Orsense-Study respectively. Similarly, the differences for anxiety were 1.00 [0.70; 1.25] (p<0.0001) and 0.8 [0.6; 1.1] (p<0.0001).

## Discussion

Compared to previous reports [4], our study showed an improvement in the accuracy of the devices with a bias close to or lower than 0.5 g/dL in both cases. However, the limits of agreement are still large, higher than 2 g/dL in both cases also.

Compared to previous reports [4], [5], [6], [7], which all studied a previous version of the same device, namely the Radical 7™ (Masimo Corporation), along with the reduction in bias, we showed several other improvements associated with this new version of the device. Concerning the monitor Pronto-7™, the repeatability of the measure is lower than previously reported but still in accordance with the required standard, fixed at 1.4% [10]. Although two factors, age and laboratory value of hemoglobin, independently affected the bias, the SpO2 is no longer associated with the bias; this constitutes a improvement compared to previous findings[4],[13]. Moreover, the rate of impossible measurement with the studied version of the device (<2.5%) is lower than previously reported (8%) [4]. Another study showed an apparent relationship between the variability of the SpHb and the perfusion index when using the Radical 7™ monitor [7], this source of variability was not present in the new version, Pronto-7™. The bias and the limits of agreement in the present study are higher than in a previously published one using the previous version of the device [6]. However, the latter involved only 35 volunteers and studied repeated measurements, whereas we chose to use one measurement per patient on a large sample. Our results on Pronto-7™ are in accordance with the results of the study conducted by Hahn et al. [5]. This study, not focused on concordance but on volume kinetic analysis of infusion fluids, reported an important variability in the non-invasive hemoglobin measurement, which differed more than 7.5% from the invasive hemoglobin in half of the paired data points. The authors concluded that non-invasive measurement of the hemoglobin concentration (using also the Radical-7™ device) during volume loading could not provide useful kinetic data for individuals [5]. Moreover, the recent study conducted by Miller et al. [7] showed limits of agreement higher than 30 g/L, similar to ours. Of note, the sample size of all previously above mentioned published works was lower than in the present study and three of those used repeated measures [5], [6], [7].

This first study of the monitor NBM-200MP™ shows a small bias but limits of agreement higher than 3 g/dl and an ICC coefficient lower than 0.8. Moreover, the measure seems to be unstable, with a mean coefficient of variation at 5.9% and an absolute difference between two consecutive measurements lower than 2.5% in less than 50% of the cases. For this monitor also, the bias is independently affected by the true level of hemoglobin and the index of perfusion is also independently associated.

The two studied monitors showed slight differences in performance. These differences could be explained by the fact that the technology used is not the same. Indeed, Pronto-7™ and NBM-200MP™ are both non-invasive solution for on-line, continuous and spot hemoglobin measurements combined with oximetry measurements, but the first uses more than seven wavelengths of light to acquire blood constituent data based on light absorption through a finger probe. The second involves a ring-shaped sensor fitted on the patient's finger that temporarily gently squeezes the finger to over-systolic pressure, similar to blood pressure measurements. Moreover, the signal processing algorithms and filters used are not the same in both cases. In contrast with these differences in performance, both devices were associated with a reduction in pain and anxiety scales with the non-invasive measure, regardless of the monitor used. Even if this result is to be expected, it is interesting for the improvement in patient care associated with the use of such point-of-care methods.

A noninvasive and accurate estimation of hemoglobin could have many roles in patient care, for example as an early warning system for bleeding, as a way of monitoring high risk patients for bleeding (for instance patients on antiplatelet therapy, cirrhotic patients, arterio-venous malformations, recurrence of peptic ulcer disease, pelvic bleeding, pregnant women at risk of placental abruption), and as a screening tool for people who have difficult venous access. It also has many theoretical advantages, such as its ability to perform repeated sampling without causing iatrogenic blood loss linked to anemia, particularly in the intensive care unit and also its ability to monitor hemoglobin concentrations in a pediatric population.

Hemoglobin can also be measured through the use of other transportable, portable, and handheld instruments at or near the site of patient care (point-of-care testing). HemoCue^®^ (HemoCue Ltd, Sheffield) provides a quick and acceptable estimation of hemoglobin compared to laboratory measurements but requires taking a blood sample[14], [15]. Interestingly, Miller et al. demonstrated that Hemocue® presented a better performance compared to non-invasive measurement using the Radical-7™ monitor from Masimo[7]. Further studies are needed to evaluate the relative performance of the two studied devices compared to HemoCue^®^.

### Limitations

Even if the two populations involved in the two studies reported in the present article seem comparable, the two devices were not compared in a single trial. Therefore, no direct comparison of their performance is possible. A comparative trial was not conducted for technical reasons, as the two devices were not available at the same time in our center. However, because of the identical design, the large sample size and the comparability of the two samples, results can be compared. The study was performed in a single center; this could limit generalization of the results. However, the large sample size led to include patients with various medical conditions. Even if the rate of anemic patients was around 30% in both studies, few patients presented extreme values of hemoglobin and the results of the studies may not be generalizable to them. Thus, further studies are needed to address this issue. Moreover, it would be interesting to test theses devices in the setting of significant fluid shifts, blood loss or active bleeding, as they are common situations occurring in the operating room, or after major trauma. The performance of the studied devices needs to be assessed in these situations.

Finally, although we studied the latest version of the device produced by Masimo, we assessed the first one produced by Orsense and another version, specifically designed for spot-check measurement, namely NBM-200™, exists but was not assessed in the present study. This could explain the slight difference between the two devices and so further investigations are needed to evaluate this alternative version.

### Conclusion

In short, two devices dedicated to non-invasive measurement of hemoglobin were assessed in the present study. Bias was found to be small but independently and inversely associated with the true value of hemoglobin. Of more importance is that limits of agreement are large in both cases making the clinical usefulness of such devices debatable.
